# Therapeutical Notes

**Published:** 1899-03-25

**Authors:** 


### THERAPEUTICAL NOTES.

### Guaiacol in the Treatment of Epididymitis.

Lenz (*Wien Klin. Rundschau*, Nos. 4—6, 1898) reports the results of administering guaiacol to 52 subjects suffering from epididymitis, of which 50 cases were due to gonorrhœa. He employs the ointment as follows:— ℞ Guaiacol, 10 parts.
Vaseline, 90 parts.
M. et signe. "For external use only."

If the skin of the scrotum is tender, the ointment should contain only 5 per cent. of guaiacol. He advises washing the skin previously with soap and ether. [Other writers have remarked that it is not desirable to apply more than 15 minims of guaiacol to the skin at once, on account of its rapid absorption and powerful effect.]—*Les Nouveaux Remedes*, July 24.

